# Lateral fluid percussion injury of the brain induces CCL20 inflammatory chemokine expression in rats

**DOI:** 10.1186/1742-2094-8-148

**Published:** 2011-10-31

**Authors:** Mahasweta Das, Christopher C Leonardo, Saniya Rangooni, Keith R Pennypacker, Subhra Mohapatra, Shyam S Mohapatra

**Affiliations:** 1 Department of Internal Medicine, University of South Florida College of Medicine, 12901 Bruce B Downs Blvd, Tampa, FL 33612, USA; 2 Department of Molecular Pharmacology and Physiology, University of South Florida College of Medicine, 12901 Bruce B Downs Blvd, Tampa, FL 33612, USA; 3 Department of Molecular Medicine, University of South Florida College of Medicine, 12901 Bruce B Downs Blvd, Tampa, FL 33612, USA; 4 JAH-VA Hospital, Tampa, FL, 13000 Bruce B. Downs Blvd. Tampa, FL 33612, USA

**Keywords:** TBI, LFPI, CCL20, inflammation, neural damage, spleen, cortex, hippocampus

## Abstract

**Background:**

Traumatic brain injury (TBI) evokes a systemic immune response including leukocyte migration into the brain and release of pro-inflammatory cytokines; however, the mechanisms underlying TBI pathogenesis and protection are poorly understood. Due to the high incidence of head trauma in the sports field, battlefield and automobile accidents identification of the molecular signals involved in TBI progression is critical for the development of novel therapeutics.

**Methods:**

In this report, we used a rat lateral fluid percussion impact (LFPI) model of TBI to characterize neurodegeneration, apoptosis and alterations in pro-inflammatory mediators at two time points within the secondary injury phase. Brain histopathology was evaluated by fluoro-jade (FJ) staining and terminal deoxynucleotidyl transferase dUTP nick end labelling (TUNEL) assay, polymerase chain reaction (qRT PCR), enzyme linked immunosorbent assay (ELISA) and immunohistochemistry were employed to evaluate the CCL20 gene expression in different tissues.

**Results:**

Histological analysis of neurodegeneration by FJ staining showed mild injury in the cerebral cortex, hippocampus and thalamus. TUNEL staining confirmed the presence of apoptotic cells and CD11b^+ ^microglia indicated initiation of an inflammatory reaction leading to secondary damage in these areas. Analysis of spleen mRNA by PCR microarray of an inflammation panel led to the identification of CCL20 as an important pro-inflammatory signal upregulated 24 h after TBI. Although, CCL20 expression was observed in spleen and thymus after 24h of TBI, it was not expressed in degenerating cortex or hippocampal neurons until 48 h after insult. Splenectomy partially but significantly decreased the CCL20 expression in brain tissues.

**Conclusion:**

These results demonstrate that the systemic inflammatory reaction to TBI starts earlier than the local brain response and suggest that spleen- and/ or thymus-derived CCL20 might play a role in promoting neuronal injury and central nervous system inflammation in response to mild TBI.

## Background

Head wounds and brain injuries following blast explosions affect more than 1.2 million Americans annually, including U.S. soldiers involved in combat operations and public safety personnel surviving terrorist attacks. It is estimated that 150-300,000 military personnel from Operation Iraqi Freedom and Operation Enduring Freedom suffered from traumatic brain injury (TBI) [1-3]

Despite the increased recognition and prevalence of TBI, the pathogenesis of TBI-induced brain injury is still poorly understood and there are currently no effective treatments. TBI is a complex process encompassing three overlapping phases: primary injury to brain tissue and cerebral vasculature by virtue of the initial impact, secondary injury including neuroinflammatory processes triggered by the primary insult, and regenerative responses including enhanced proliferation of neural progenitor cells and endothelial cells. Therapies aimed at reducing TBI injury must be focused on blocking the secondary inflammatory response or promoting regeneration and repair mechanisms.

The secondary damage is progressive, evolving from hours to days after the initial trauma, and is largely due to injury of the cerebral vasculature. Degradation of the blood brain barrier (BBB) permits extravasation of circulating neutrophils, monocytes and lymphocytes into the brain parenchyma [4-6]. Inflammatory factors released by these infiltrating immune cells as well as resident microglia can cause cell death. Also, multi-organ damage in trauma patients can lead to elevated circulatory levels of inflammatory cytokines that may contribute to the post-TBI pathogenesis of the brain [7]. Spleen, a reservoir of immune cells, plays an important role in initiating the systemic ischemic response to stroke and neurodegeneration [8]. Reduction in splenic mass with corresponding increase of immune cells in circulation following TBI has been observed recently by Walker et al. [9]. Various cytokines and chemokines have been reported to be involved in TBI, including IL-1, IL-6, IL-8, IL-10, granulocyte colony-stimulating factor, tumour necrosis factor-α, FAS ligand and monocyte chemo-attractant protein 1 [7,10] and are thought to account for the progressive injury. But, there is a paucity of mechanistic data implicating activated microglia, reactive astrocytes, or peripheral leukocytes in the release of inflammatory molecules that exacerbate TBI injury.

While profiling of inflammatory markers provides some clues regarding the source and progression of TBI pathology, it has not led to the development of a successful therapy to combat TBI-induced brain damage and its long term outcome. Therefore, identification of one or more specific molecules as unique biomarkers and therapeutic targets is of critical importance in extending experimental treatments to patients. The present study was conducted to examine the relationship between the brain response to TBI and the systemic immune response in a rat model of TBI. The LFPI model of TBI used in this study offers an excellent model of clinical contusion without skull fracture [11,12], expressing the features of the primary injury including the disruption of the BBB, secondary injury and diffuse axonal injury [13]. In this study, we characterized the injury caused by LFPI in the rat and identified CCL20 as both a peripheral and local immune signal in the pathogenesis of TBI.

## Methods

### Animals

All animal procedures were conducted in accordance with the NIH Guide for the Care and Use of Laboratory Animals following a protocol approved by the Institutional Animal Care and Use Committee at the University of South Florida. Male Sprague-Dawley rats (Harlan, Indianapolis, IN) weighing 250 to 300 g were housed in a climate-controlled room with water and laboratory chow available *ad libitum*. A total of 33 animals were used in this study.

### Induction of Lateral Fluid Percussion Injury (LFPI)

Animals were anesthetized using a mixture of ketamine (90 mg/kg)/xylazine (10 mg/kg) (IP). To deliver LFPI, a 1 mm diameter craniotomy was performed centered at 2 mm lateral and 2.3 mm caudal to the bregma on the right side of the midline. A female luer-lock hub was implanted at the craniotomy site and secured with dental cement. The FPI device was then fastened to the luer-lock. All tubing was checked to ensure that no air bubbles had been introduced, after which a mild impact ranging from 2.0-2.2 atm. was administered [14]. Impact pressures were measured using a transducer attached to the point of impact on the fluid percussive device. The luer-lock was then detached, the craniotomy hole was sealed with bone wax and the scalp was sutured. Ketoprofen (5 mg/kg) was administered to minimize postsurgical pain and discomfort. Rats were then replaced in their home cages and allowed to recover for 24-48 h prior to subsequent experiments. Animals were excluded from further tests if the impact did not register between 2.0 and 2.2 atm. or if the dura was disturbed during the craniotomy prior to impact. In sham (control) animals, craniotomy was performed at the same coordinates as the TBI animals but no impact was delivered.

#### Splenectomy

To remove the spleen from the anesthetized rat a cranial-caudal incision was made lateral to the spine with the cranial terminus of the incision just behind the left rib cage. A small incision was made on the exposed muscle layer to access the spleen. The spleen was then pulled out through the incision, the splenic blood vessels were tied with 4.0 silk sutures and the spleen was removed by transecting the blood vessels distal to the ligature. The attached pancreatic tissues were detached from the spleen by blunt dissection and returned to the abdominal cavity before removal of the spleen. The muscle and skin incisions were sutured and the animals were allowed to survive for 24 or 48 hours.

### Tissue collection

Animals were deeply anesthetized with ketamine (75 mg/kg) and xylazine (7.5 mg/kg) 24 or 48 hours after TBI. Thymuses and spleens were removed and immediately snap frozen on dry ice. Animals were then perfused with 0.9% saline followed by 4% paraformaldehyde in phosphate buffer (pH 7.4). The brains were harvested, post-fixed in 2% paraformaldehyde and saturated with increasing sucrose concentrations (20% to 30%) in phosphate-buffered saline (PBS, pH 7.4). Brains were then frozen, sectioned coronally at 30 μm thickness using a cryostat, thaw-mounted onto glass slides and stored at -20°C prior to staining. In the initial studies 80% of the injured neurons were found in the brain region between 3.5 and 5.5 mm caudal to the bregma. Therefore, for all subsequent staining experiments, three sections from each brain corresponding to 3.5, 4.5, and 5.5 mm caudal to the bregma were selected for analysis.

### RNA extraction, purification and cDNA synthesis

Total RNA was extracted from 50 mg of frozen spleen tissue using TRIZOL reagent (Invitrogen, Carlsbad, CA). Briefly, the samples were homogenized with 1 ml of TRIZOL, incubated at room temperature for 5 minutes and phase-separated by chloroform. Total RNA was precipitated by isopropyl alcohol, collected by centrifugation and purified using an RNeasy mini kit (Qiagen, Valencia, CA). The RNA concentration and purity was determined by spectrophotometry at 260/280 nm and 260/230 nm. First strand cDNA was synthesized from the isolated RNA using the Superscript III system (Invitrogen).

### mRNA SuperArray analysis

A panel of proinflammatory cytokines and chemokines and their receptors was analyzed using a SYBR green-optimized primer assay (RT^2 ^Prolifer PCR Array) from SA bioscience (Frederick, MD). Briefly, cDNA was synthesized from fresh frozen spleens as stated above. cDNA was mixed with the RT2 qPCR master mix and the mixture was aliquoted across the PCR array. The PCR was done in a CFX96 Real-Time C1000 thermcycler (BioRad) for 5 min at 65C, 50 min at 50C and 5 min at 85C. Control gene expression was normalized and target gene expression was expressed as fold increase or decrease compared to control. PCR data were analyzed using the SA Bioscience Excel program.

### Enzyme-linked immunosorbent assay (ELISA) for CCL20

Spleen tissue lysates were prepared from 5 mg of fresh frozen tissue using protein lysis buffer containing NP-40. CCL20 was estimated by ELISA using the DuoSet ELISA Development kit for CCL20 from R & D systems (Minneapolis, MN). Briefly, 96 well sterile ELISA microplates were coated with anti-rat CCL20α antibody overnight at room temperature. Next day, the plates were washed and blocked with bovine serum albumin (BSA). Plates were incubated sequentially with standards or samples for 2 h, detection antibody (biotinylated goat anti-rat CCL20α antibody) for 2 h, streptavidin-HRP for 20 minutes and substrate solution (1:1 mixture of H_2_O_2 _and tetramethylbenzidine) for 20 minutes. Reactions were stopped with 2N H_2_SO_4_. All incubations were performed at room temperature and the microplate was thoroughly washed after each incubation. The absorbance of each well was determined at 450 nm using a Synergy H4 Hybrid reader (BioTek). Total protein concentrations from the same samples were determined by BCA protein assay (Pierce). CCL20 was expressed as pg per μg of total protein in the tissue.

### Fluoro-Jade histochemistry

Fluoro-Jade (Histochem, Jefferson, AR) staining was performed to label degenerating neurons. This method was adapted from that originally developed by Schmued et at [15] and subsequently detailed by Duckworth [16]. Thaw-mounted sections were placed in 100% ethanol for 3 minutes followed by 70% ethanol and deionized water for 1 minute each. Sections were then oxidized using a 0.06% KMnO_4 _solution for 15 minutes followed by thee rinses in ddH2O for 1 minute each. Sections were then stained in a 0.001% solution of Fluoro-Jade in 0.1% acetic acid for 30 min. Slides were rinsed, dried at 45°C for 20 min, cleared with xylene, and cover-slipped using DPX mounting medium (Electron Microscopy Sciences, Ft. Washington, PA).

### TUNEL staining

Nuclear DNA fragmentation, a marker of apoptotic cells was measured using the DeadEnd Fluorimetric TUNEL system (Promega, Madison, WI). Fixed cryosections (30μ thick) were permeabilized with 20 μg/ml proteinase K at room temperature for 8 minutes followed by 4% PFA in PBS for 5 minutes. The sections were washed in PBS and equilibrated with 200 mM potassium cacodylate, pH 6.6; 25 mM Tris-HCl, pH 6.6; 0.2 mM DTT; 0.25 mg/ml BSA and 2.5 cobalt chloride (equilibration buffer) for 10 minutes at room temperature. The sections were then incubated at 37°C for 1 hour with incubation buffer containing equilibration buffer, nucleotide mix and rTdT enzyme mix, covered with plastic cover slip and placed away from exposure to light. The cover slips were removed and the reactions were stopped with 2X SSC. The sections were then washed with PBS and mounted with VectaShield mounting medium containing DAPI. The green fluorescence of fluorescein-12-dUTP was detected in the blue background of DAPI under the fluorescence microscope. Images were taken and apoptotic nuclei were quantified using the Image J quantitation program.

### Immunohistochemistry

Spleen, thymus or brain tissue sections were washed with PBS for 5 min, incubated in 3% hydrogen peroxide for 20 min and washed 3 times in PBS. They were then heated in antigen unmasking solution (1:100; Vector Laboratories Inc., Burlingame, CA) for 20 min at 90°C, incubated for 1 h in permeabilization buffer (10% goat serum, 0.1% Triton X-100 in PBS) and incubated overnight at 4°C with either rabbit anti-CCL20 primary antibody (1:1000) or mouse monoclonal anti-CD11b antibody (1:400) (Abcam, Cambridge, MA) in antibody solution (5% goat serum, 0.05% Triton X-100 in PBS). The following day, sections were washed with PBS and incubated 1 h at room temperature with secondary antibody (biotinylated goat anti-rabbit, 1:400, Vector Laboratories Inc., Burlingame, Ca or Alexafluor 594 conjugated antimouse antibody, 1:50 or DyLight 594 conjugated antirabbit antibody, 1:50) in antibody solution. Sections incubated with biotinylated antirabbit antibody were then washed in PBS, incubated in avidin-biotin complex mixture (ABC,1:100; Vector Laboratories Inc, Burlingame, Ca) for 1 h, washed again and visualized using DAB/peroxide solution (Vector Laboratories Inc). After three washes, sections were dried, dehydrated with increasing concentrations of ethanol (70%, 95%, 100%), cleared with xylene and cover-slipped with Vectamount mounting medium. Sections incubated with mouse anti-CD11b antibody followed by alexafluor 594-conjugated antimouse antibody were washed three times with PBS and used for double staining with IB4. Some of the anti-CCL20 antibodies followed by DyLight 594-conjugated antirabbit antibody treated sections were incubated with Alexa fluor 488-conjugated mouse antineuronal nuclei (NeuN) monoclonal antibody (1:100; Millipore, Temecula, CA) 3 hours at room temperature, washed with PBS, dried and cover slipped with vectamount mounting medium with DAPI.

### CCL20 - Fluoro-Jade double staining

Slide mounted sections were washed in PBS and CCl20 immunostaining was performed as described above and developed with DyLight 594 conjugated anti rabbit antibody. Sections were then incubated in acidic 0.0001% FJ solution for 20 min on shaker. Slides were washed, dried and cover slipped with Vecta Shield mounting medium.

### Isolectin IB4 histochemistry

Brain sections were washed with modified PBS (PBS with 0.5mM CaCl_2_, pH 7.2) and permeabilized with buffer containing 10% goat serum, 3% lysine, 0.3% triton X-100 in modified PBS for 1 hour at room temperature. Brain sections already immunostained were transferred to modified PBS. Sections were then incubated overnight at 4°C with 5 μg/ml Alexa 488-conjugated isolectin IB4 (Molecular Probes) dissolved in modified PBS with 0.3% triton X-100 and 2% goat serum. Stained sections were washed with modified PBS, mounted with Vecta-Shield mounting medium with DAPI and viewed with an Olympus IX71 fluorescent microscope using the FITC filter. Images were taken using the Olympus DP70 imaging system and IB4-positive cells were quantified using the Image J quantitation program.

### Image analysis and quantitation

All quantitation was performed using the NIH Image J software. For immunohistochemical analysis, images were acquired using an Olympus IX71 microscope controlled by DP70 manager software (Olympus America Inc., Melville, NY). Photomicrographs captured at 200x magnification with an Olympus DP70 camera were used for quantification. Images were taken at the same exposure and digital gain settings for a given magnification to minimize differential background intensity or false-positive immunoreactivity across sections. The channels of the RGB images were split and the green channel was used for quantitation of the FJ, IB4 and TUNEL staining images. The CCL20 images were converted to gray-scale before quantitation. The single channel or gray-scale images were then adjusted for brightness and contrast to exclude noise pixels. The images were also adjusted for the threshold to highlight all the positive cells to be counted and a binary version of the image was created with pixel intensities 0 and 255. Particle size was adjusted to exclude the small noise pixels from the count. Circularity was adjusted to between 0 and 1 to discard any cell fragments, processes or tissue aggregates resulting in false labelling from the quantitation. The same specifications were used for all sections. Cell counts of sections from 3.5, 4.5 and 5.5 mm caudal to the bregma were summed to represent the number of positive cells from each brain. The results for the FJ, TUNEL, IB4 and CCL20 immunoreactivity were expressed as mean number of positive cells ± S.E.M. CCL20 immunoreactivity of the thymus or the spleen was expressed as mean area of immunoreactivity ± S.E.M.

### Statistical analysis

All data are presented as mean ± S.E.M. Statistical significance was evaluated by one-way ANOVA with Bonferroni's post-hoc test. A *p *value of less than 0.05 was considered statistically significant for all comparisons.

## Results

### Regional distribution of neurodegeneration after TBI

**Figure 1:**
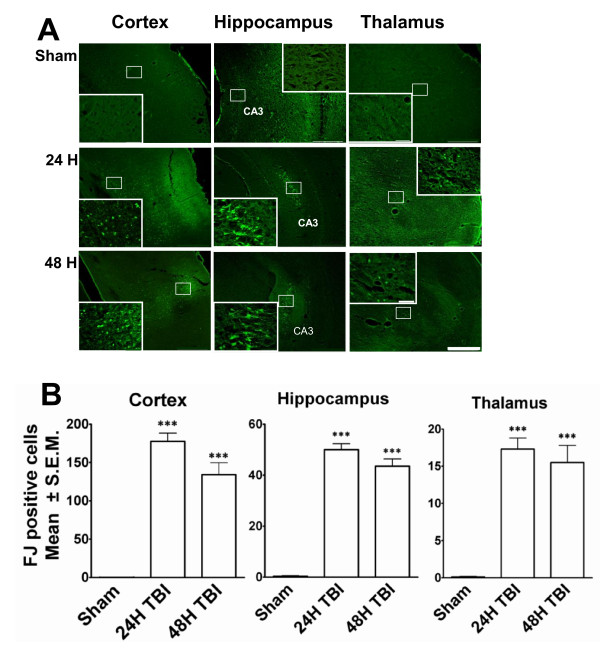
**TBI induces neurodegeneration in different areas of the rat brain**. Fluoro Jade (FJ) staining was performed on cryosections from rat brains to identify the damaged neurons 24 hours or 48 hours after the induction of mild lateral fluid percussion impact (LFPI). **A**. Representative low magnification (40X) photomicrographs showing FJ-positive neurons indicating neurodegeneration in cortex (left column), hippocampus (middle column) and thalamus (right column) 24 hours or 48 hours after LFPI. No degenerating neurons were observed in the corresponding brain regions in the sham animals. Scale bar = 500 μ. High magnification (400X) images from selected areas of respective sections are shown in the inset. Scale bar = 50 μ. **B**. The FJ-positive neurons were quantitated using the Image J program. The histograms show the estimation of FJ-positive neurons in cortex, hippocampus and thalamus. Cortex showed the highest number of injured neurons compared to other regions. Most FJ-positive neurons were observed after 24 hours of injury in all three regions. The numbers of degenerating neurons went down 48 hours after TBI but were significantly higher compared to sham animals. *** p < 0.001 compared to sham animals.

Inconsistencies in injury assessment across laboratories and lack of a reliable, quantitative approach to assessing neural injury have impeded efforts to develop novel treatments for TBI pathology. Therefore, a detailed investigation throughout the brain was sought to determine which regions show consistent, prominent neurodegeneration in rats subjected to mild LFPI (Figure 1). A consistent profile emerged in which the majority of Fluoro-Jade (FJ)-positive cells were found within the cerebral cortex (Figure 1), hippocampus (Figure 1), and thalamus (Figure 1). Cortical Fluoro-Jade was ubiquitous and was present at various levels throughout the brain. Hippocampal FJ staining was localized to the pyramidal cell layers (Figure 1), while some diffuse labelling throughout the general structure was also evident. The thalamic staining was diffuse and sparsely distributed. Quantitation revealed that the neurodegeneration in these regions significantly increased at both 24 and 48 h post-impact relative to sham-operated controls. Additionally, data showed that FJ-stained degenerating hippocampal neurons were restricted to the ipsilateral hemisphere, whereas few cortical and thalamic FJ-positive neurons were also detected in the contralateral hemisphere in some animals.

### Mild TBI-induced internucleosomal DNA fragmentation in the cortex and hippocampus

**Figure 2:**
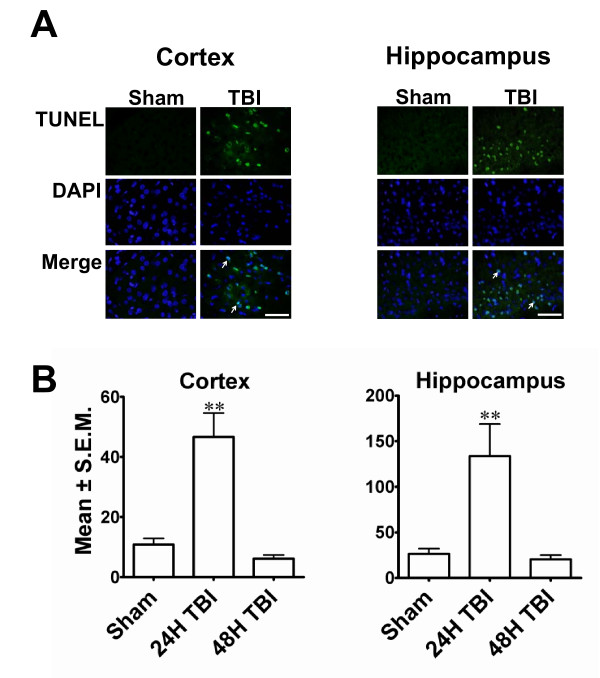
**TBI causes DNA damage 24 hours after impact**. **A**. Photomicrographs of representative sections from rat cortex or hippocampus showing TUNEL histochemistry 24 hours after mild LFPI. TUNEL-positive nuclei (green fluorescence) were distributed throughout the ipsilateral cortex or hippocampus 24 h after TBI. Intense signals are observed as rims on the nuclear boundaries with a diffuse homogeneous signal on the interior of the nucleus. Arrows indicate the TUNEL positive nuclei. (Scale bar 500 μ). **B**. Histograms show the number of TUNEL-positive nuclei in the cortex or hippocampus 24 or 48 hours after TBI. Significant increase in the TUNEL-positive nuclei at the 24 h time point indicates the DNA damage occurs in these brain regions as early as 24 hours post-TBI although at 48 hours after TBI the damage was not significantly different in TBI animals compared to sham-treated animals. (** *p *< 0.001 compared to sham animals)

Internucleosomal DNA fragmentation, an important marker for apoptotic cells, was assessed by terminal deoxynucleotidyl transferase biotin-dUTP nick end labelling (TUNEL) histochemistry. Few TUNEL-positive cells were detected in the contralateral hemisphere, and, while the ipsilateral thalamus showed sparse TUNEL staining in some sections, this was not a consistent finding throughout the experiment (data not shown). The majority of TUNEL-stained nuclei were detected at 24 h post-TBI in the ipsilateral cortex (Figure 2A) and hippocampus (Figure 2B), while sections from sham-operated controls were predominantly devoid of TUNEL staining in these regions (Figure 2A, Figure 2B) and showed only background levels of fluorescence. By 48 h after TBI, sections showed very few TUNEL-positive cells in the cortex and hippocampus and resembled sham-operated controls. Quantitation revealed a significant increase in TUNEL-positive cells in both cortex and hippocampus 24 h post TBI as compared to sham-operated control groups (Figure 2C).

### Microglia are activated in the brain following mild TBI

**Figure 3:**
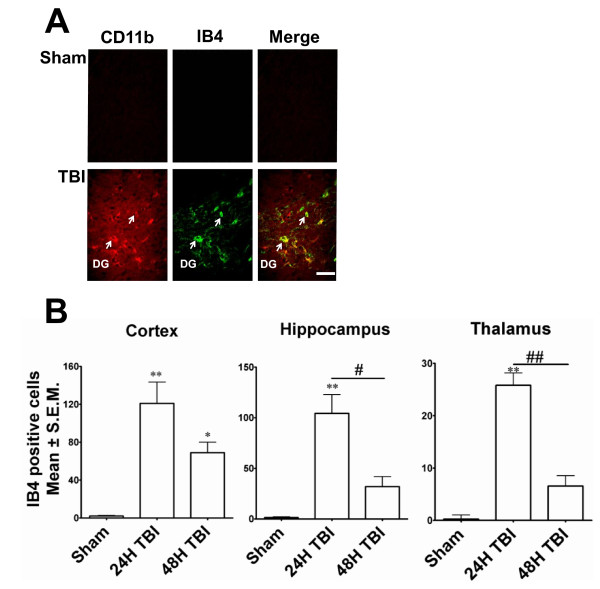
**Mild TBI activates microglia 24 hours after impact**. IB4-positive cells were observed in different areas of brain 24 hours after TBI. Some of these cells were CD11b-positive. This labelling was absent in the sham animals and significantly less on the contralateral side or 48h after TBI. **A**. Confocal microscopic images showing IB4-positive (Alexafluor 488-conjugated, green fluorescence), CD11b-positive (red fluorescence) or IB4/CD11b-positive (red-green overlap) microglia in representative sections of ipsilateral dentate gyrus 24 hours after moderate TBI. The left column shows CD11b immunostaining, the middle column IB4 labelling and the right column is an overlay of CD11b and IB4 double labelling. Arrows indicate the CD11b or IB4 or CD11b-IB4 positive cells. Scale bar 30μ.**B**. Histograms show the quantitation of IB4-positive microglia in the ipsilateral cortex, hippocampus and thalamus 24 or 48 hours after TBI. In all three regions, the number of IB4-positive cells was significantly increased 24 h after TBI compared to sham animals. ** p < 0.001; * p < 0.05; compared to sham; # p < 0.05, ## p < 0.001 compared to 24H TBI.

Isolectin-IB4, a 114 kD protein isolated from the seeds of the African legume, *Griffonia simplicifolia *has been shown to have a strong affinity for resident microglia in the central nervous system and peripheral macrophages that are activated in response to neural injury. To assess the local inflammatory response following mild TBI, Alexa-Fluor 488-conjugated IB4 was used to label microglia/macrophages in the brain tissue. While IB4 labelling was primarily restricted to the ipsilateral hemisphere, sparse labelling was detected within the contralateral hippocampus (data not shown). IB4-positive cells were abundant in the hippocampus, especially in the dentate gyrus (Figure 3A). Microglia were also found in the cortex and thalamus (data not shown) following TBI. CD11b, an activated microglial marker, was also found in the cells of the cortex and hippocampus (dentate gyrus, Figure 3A) of the ipsilateral side. Confocal microscopy revealed that most but not all IB4^+ ^cells in the cortex or hippocampus were also CD11b^+ ^(Figure 3A). Quantitation showed that the number of IB4-positive cells was significantly increased in each of these brain regions 24 h after TBI, while number of IB4^+ ^cells in these regions 48 h post-TBI did not significantly differ from sham-operated controls (Figure 3B). These observations indicate that an inflammatory response was mounted within the brain parenchyma as early as 24 h after the injury involving microglial activation/ migration to the site of injury.

### CCL20 is identified as a major inflammatory gene expressed in the spleen and thymus following TBI

**Figure 4:**
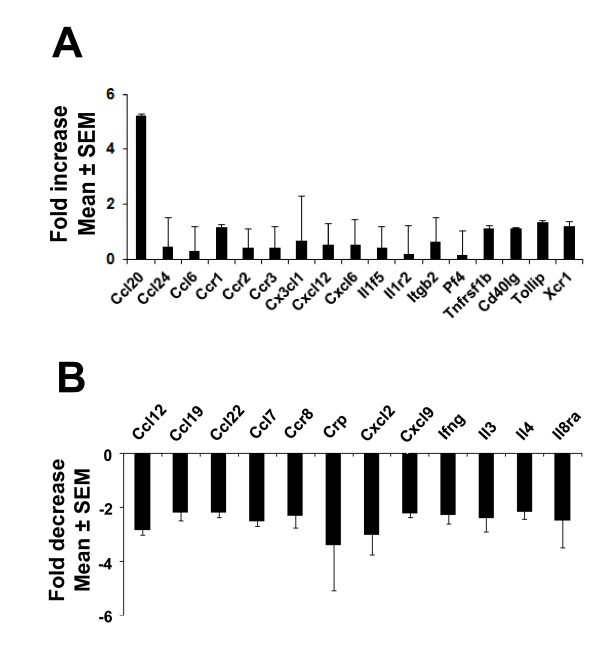
**CCL20 is up-regulated in spleen 24 hours after mild TBI**. PCR super array analysis was performed to analyze the gene expression in spleen tissues following TBI. The histograms show the mRNA expressional changes of different cytokines, chemokines and their receptors 24 hours after TBI. **A: **The up-regulated genes: CCL20 mRNA increased 5-fold in TBI animals compared to the sham animals. **B: **The down-regulated genes with 2-fold or more down-regulation.

**Figure 5:**
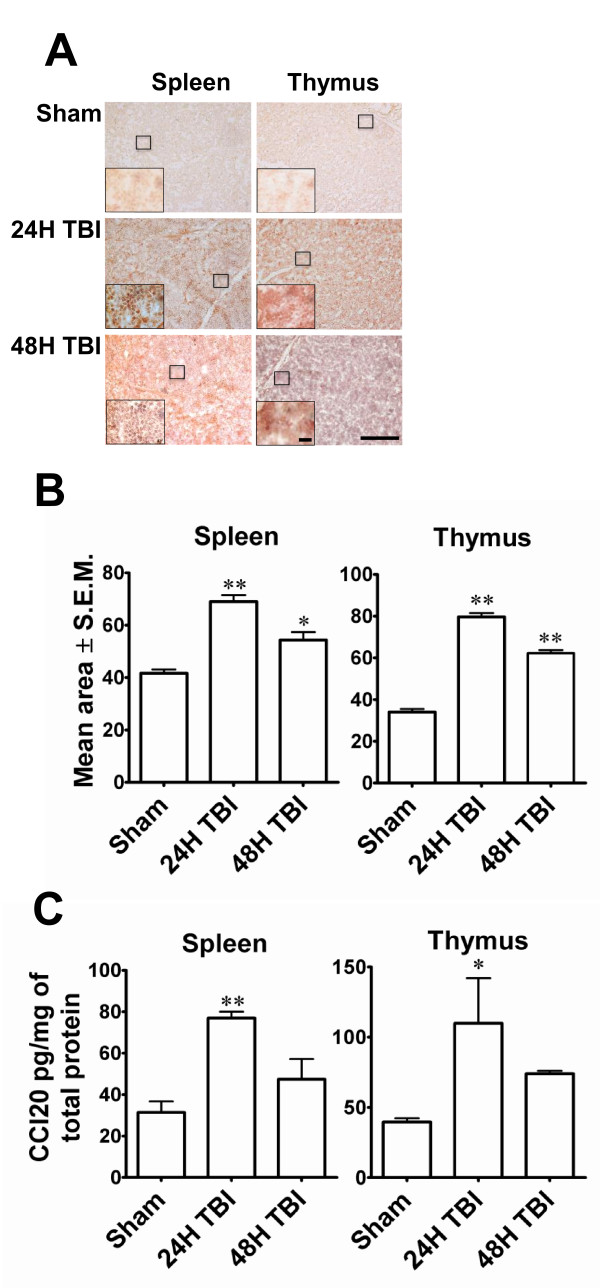
**CCL20 expression is up-regulated in spleen and thymus after mild TBI**. **A: **Low magnification (scale bar 500 μ) photomicrographs showing the immunohistochemical labelling of CCL20 in spleen and thymus tissues in sham, and 24 h or 48 h after TBI. High magnification (scale bar 20 μ) images of the selected areas from each section are shown in the inset of the corresponding image. **B**. CCL20 immunoreactivity in spleen or thymus in sham or TBI animals was quantitated using the Image J program and expressed as mean area ± S.E.M. CCL20 immunoreactivity increased significantly 24 h and 48 h after TBI compared to sham animals. *p < 0.05, **p < 0.001 compared to sham. **C**. The histograms show the changes of CCL20 expression in spleen and thymus 24 or 48 hours post TBI. ELISA was performed with rat anti-CCL20 antibody using a Duo set ELISA kit from R&D systems. In both tissues CCL20 expression increased significantly 24 h after TBI. *p < 0.05, ** *p *< 0.001 compared to sham animals.

Several studies have suggested that in addition to the local response, activation of the systemic inflammatory response is critical in inducing TBI-associated neuropathies. Although a number of cytokines and chemokines have been studied, the key systemic inflammatory molecules have not yet been identified. Because the spleen has been shown to be involved in the systemic inflammatory response in various injury models, SuperArray analysis was performed on spleen RNA from three separate experiments to identify alterations in the expression of genes associated with pro-inflammatory signalling after LFPI (Figure 4). SuperArray data indicates that more genes were down-regulated (Figure 4B) than were up-regulated (Figure 4A). Among the genes that were up-regulated, CCL20 was uniquely up-regulated by five-fold compared to controls (Figure 4A) 24 h after TBI. These studies led to the identification of CCL20 as a potentially important pro-inflammatory, systemic marker of TBI. To confirm this observation as well as to determine whether alterations in CCL20 mRNA paralleled protein expression, ELISAs and immunohistochemistry were performed on spleen tissues. Immunohistochemistry on spleen tissues indicated significant up-regulation of CCL20 expression at 24 h after TBI as indicated by the increase in mean area of CCL20 intensity. Significant expression of the protein was also observed 48 h after impact (Figures 5A, B). The immunohistochemical observation was further supported by the data obtained from ELISA of spleen tissues showing at least two-fold up-regulation of CCL20 protein expression 24 h after TBI (Figure 5C). In addition to spleen, the thymus also expressed CCL20 at 24 h after TBI as evident from the immunohistochemical labelling of thymus (Figure 5A and 5B) and ELISA for CCL20 of thymic tissues (Figure 5C). These observations support the notion that CCL20 chemokine signalling contributes to the systemic inflammatory response, and that the spleen and thymus respond as early as 24 h after TBI.

### CCL20 is expressed in the brain following TBI-induced neurodegeneration

**Figure 6:**
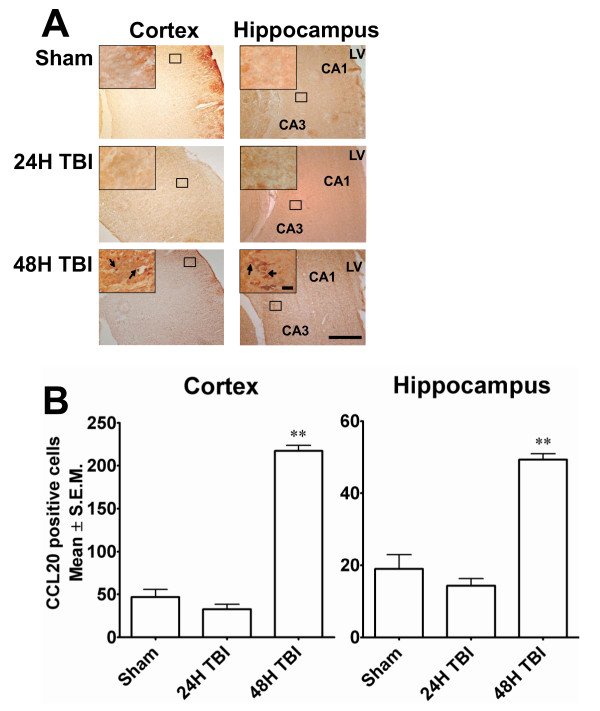
**CCL20 is expressed in rat brain cortex and hippocampus 48 h after TBI**. **A**. Immunostaining with anti CCL20 antibody shows CCL20-expressing cells in cortex and hippocampus 48 h after TBI. Low magnification (scale bar 500μ) photomicrographs with high magnification (scale bar 50μ) images from selected areas are shown in the inset. The immunostaining was localized in the pyknotic cell bodies (arrows) devoid of surrounding tissues indicating tissue damage. This immunostaining was not evident 24 h after TBI. Arrows indicate the CCL20-expressing cells. **B**. CCL20-positive neurons in ipsilateral cortex and hippocampus were counted using the NIH Image J program and compared with corresponding areas from sham animals. CCL20 expression significantly increased in TBI animals 48 hours after impact. **p < 0.001 compared to sham.

Data from the regional injury distribution experiments showed that mild TBI resulted in highly reproducible cellular injury within the cortex as well as the hippocampus. Because splenic CCL20 expression was increased in the acute phase of TBI injury (24 h post-insult) and the splenic inflammatory response is known to exacerbate neural injury [10,17,18] experiments were performed to determine whether CCL20 expression is associated with neural injury. Brain sections from animals subjected to mild TBI or sham-TBI were immunostained for CCL20 expression using an antibody generated against the same CCL20 antigen that was used to immunostain the spleen and thymus sections (Figure 6).

**Figure 7:**
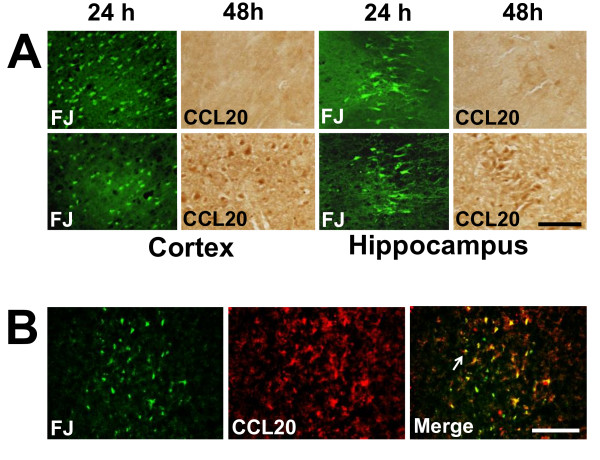
**CCL20 expression is observed in the areas of neurodegeneration of cortex and hippocampus 48 hours after TBI**. **A**. High magnification photomicrographs of brain sections from animals subjected to TBI and sacrificed 24 or 48 h post-impact were stained with Fluoro-Jade or anti-CCL20 antibody. Fluoro-Jade staining was observed in the cortex and in the hippocampal CA1 and CA3 pyramidal cell layers 24 and 48 hours after TBI. While no CCL20 immunoreactivity was observed in the same regions of adjacent sections 24 h after TBI, CCL20 immunoreactivity was observed in the cortical neurons as well as within the hippocampal CA1 and CA3 pyramidal cell layers at 48 h. FJ, Fluoro Jade. Scale bar 50μ. **B**. Representative photomicrographs showing the FJ - CCL20 double staining in the cortex. CCL20 immunoreactivity was observed in most of the degenerating neurons (FJ positive) as indicated by arrows. CCL20 immunoreactivity was also observed in other cells those were not FJ positive. Scale bar 100μ.

**Figure 8:**
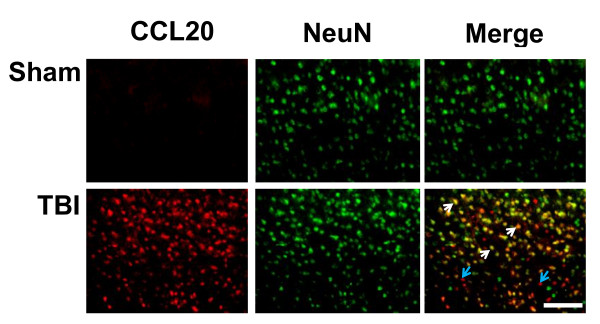
**CCL20 is expressed in rat brain cortical neurons 48 h after TBI**. Fluorescence microscopic images double immunostained with anti CCL20 antibody and the neuronal marker NeuN antibody showed most of the CCL20-expressing cells in the cortex were also NeuN positive. White arrows indicate CCL20 positive neurons, blue arrows indicate CCL20 positive non neuronal cells. Scale bar 100 μ.

CCL20 immunoreactivity was observed in the cortex and hippocampus 48 h after TBI. In the cortex CCL20 was expressed in the ipsilateral as well as contralateral sides. The immunoreactivity was observed in the CA1 and CA3 hippocampal pyramidal cell layers and was restricted to ipsilateral side of the brain. CCL20 immunoreactivity was absent in the 24 h group. Additionally, CCL20-positive neuronal cell bodies displayed pyknotic morphology and were surrounded by areas devoid of tissue (Figure 6A; Figure 7A). The immunohistochemical observation was further supported by the quantitation of the CCL20-positive cell bodies which showed a significant increase in CCL20-positive neurons in the cortex and hippocampus of rats euthanized 48 h post-TBI compared to 24 h or sham control rats (Figure 6B). It is noteworthy that although CCL20 immunoreactivity was not seen in the damaged neurons at 24 h, it was expressed by the neurons of cortex and hippocampus (Figure 7A), including the degenerating ones in these regions at 48 h after impact as evident by the co-localization of FJ and CCL20 stainings (Figure 7B). Importantly, CCL20 expressing cells in the cortex (Figure 8) and hippocampus (data not shown) were mostly neurons as they were also NeuN positive. Taken together, these observations demonstrate that CCL20 expression is increased in the brain due to TBI-induced neuronal injury at a later time point than the systemic increase of the same chemokine in response to mild TBI and may play a role in the neural injury and inflammatory reaction in the brain.

### Splenectomy attenuates TBI-induced neurodegeneration and CCL20 expression in the cortex

**Figure 9:**
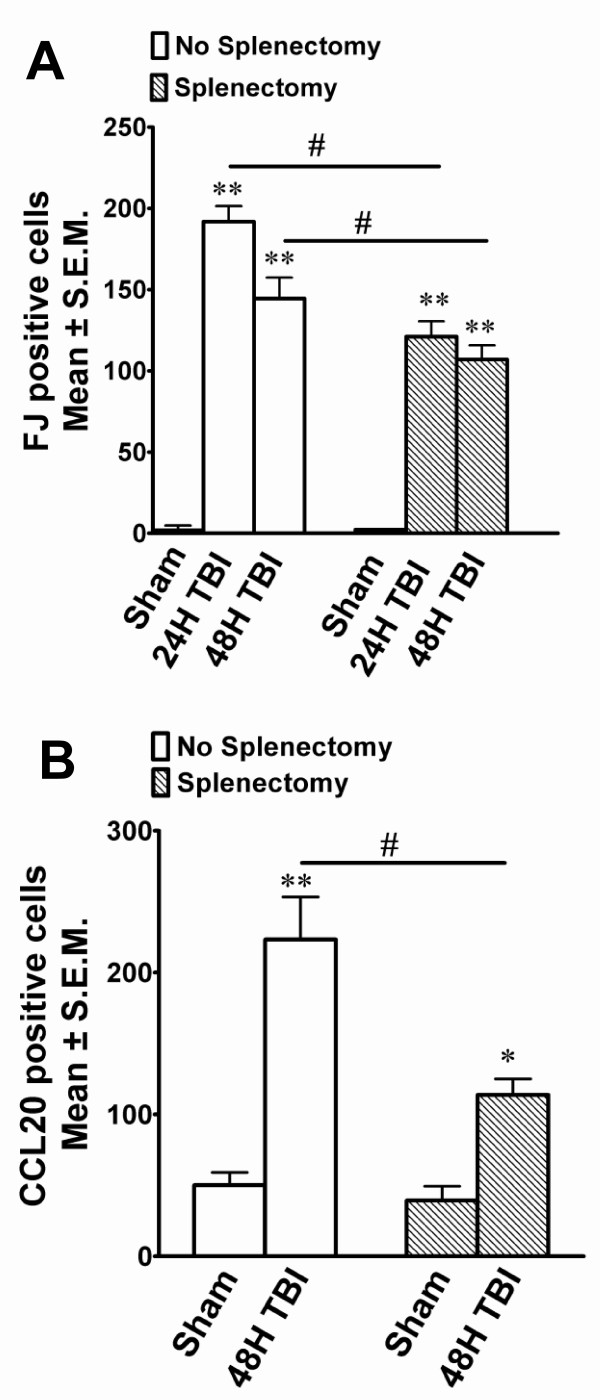
**Immediate splenectomy reduces TBI-induced neurodegeneration and CCL20 expression in the cortex**. Degenerating neurons (FJ positive) were observed 24 hours or 48 hours after the induction of LFPI in animals with (splenectomy group) or without (no splenectomy group) immediate splenectomy. **A**. The histograms show the estimation of FJ-positive neurons as quantitated by the Image J program in the cortex. **B**. CCL20 expression was observed in the cortex 48 hours after LFPI in animals with (splenectomy) or without (no splenectomy) immediate splenectomy. The histograms show the estimation of CCL20-positive cells in the cortex. ** p < 0.0001 and * p < 0.001 compared to sham animals within the group. # p < 0.001 compared to 24h or 48h TBI between the groups.

To evaluate the significance of the spleen in LFPI-induced neurodegeneration, splenectomy was performed immediately after the induction of TBI. FJ histochemistry and CCL20 immunostaining were performed to evaluate the extent of damage in splenectomised animals. It was observed that in splenectomised rats the number of FJ-positive cells was significantly reduced compared to non-splenectomised animals at the same time points, while within the splenectomy group the number of FJ-positive cells was significantly increased after TBI compared to splenectomised shams (Figure 9A). Splenectomy also reduced CCL20 expression in the cortex 48 h after TBI. In splenectomised rats, CCL20 expression increased significantly when compared to splenectomised sham animals; but the CCL20 expression was reduced significantly when the spenectomised TBI rats were compared to the non-splenectomised TBI group. These observations indicate that the spleen plays a role in TBI induced neurodegeneration and CCL20 expression in the rat brain after mild TBI.

## Discussion

Mild TBI comprises almost 80% of clinical TBI. Despite continuing research and accumulated knowledge, an effective treatment for mild TBI is still not available. In the present study, we have adopted the LFPI model of TBI originally characterized by McIntosh et. al. [19] to develop a methodology that results in quantifiable reproducible injury. Because pressure pulses within the range used here (2.0-2.2 atm.) are generally considered to reflect mild injury in the rat model [14], this paradigm is particularly attractive in that it lends relevance to the clinical population suffering from mild injury. However, conflicting data in the literature regarding the regional and temporal injury distribution prompted us to conduct a comprehensive investigation throughout the brain to determine where approximately 80% of the injury is found at 24 and 48 h post-impact. These endpoints were selected because they represent a delayed window within the secondary injury phase that can be targeted by novel therapeutics.

Consistent with previous studies using the LFPI [20,18,21] FJ and TUNEL staining in this study showed that the predominant areas of neurodegeneration and apoptosis include the cerebral cortex, hippocampus, and thalamus. In a previous study using similar TBI methodology, Sato et al. [21] showed Fluoro-Jade and TUNEL staining that persisted from 3 hours to 7 days and included cerebellar damage in addition to damage in those regions identified here. Furthermore, we have demonstrated that LFPI-induced neurodegeneration paralleled with increase in activated microglia in the injured brain. Also, in accordance with the observation of Stahel et al., the dying neurons showed characteristics of both necrosis and apoptosis [22]. These observations indicate that substantial inflammation takes place in the brain parenchyma in response to mild LFPI in this study.

The nature and progression of TBI-induced brain pathology limits the goals of treatment to either blocking the secondary injury phase or facilitating plasticity and repair at some point after the initial impact. The secondary phase is largely a result of the migration of activated microglia towards the site of injury, secreting toxic cytokines and oxygen radicals and thereby causing further neuronal damage [13,23,24]. Lunemann et. al. [25] have shown that following the formation of a brain lesion, microglia invade the damaged brain tissue after maturing and becoming activated by producing macrophage activating factor (MAF) in a CD11b-positive pathway. In agreement with this finding, we have also observed that within 24 h of initial damage the brain parenchyma is invaded by activated microglial cells. This indicates that an active inflammatory reaction is generated locally in the brain as early as 24 hours after injury.

The spleen is a reservoir of peripheral macrophages and other immune cells in the body, and it is now well known that splenic signalling contributes to injury of various tissues after ischemic insult. For example, splenectomy prior to insult protects both the liver [26] and brain [8] from ischemic damage. In a recent study, Li et al. have shown that splenectomy immediately after TBI in rats decreased[18] proinflammatory cytokine production and mortality rate and improved cognitive function. In our study, we observed that splenectomy immediately after induction of TBI attenuated TBI-induced neurodegeneration and CCL20 expression in the brain. Although it is not clear how this spleen-brain interaction takes place, Lee et al. [27] suggested that vagal nerve stimulation may reduce immune cell infiltration and consequent decrease in brain inflammation and edema while Stewart and McKenzie [28] suggested a role of sympathetic stimulation in causing the release of immune cells from spleen and subsequent infiltration into the brain tissues. Regardless of the neural mechanism, removal of the spleen immediately after the insult would remove the largest pool of immune cells, resulting in decreased infiltration and consequent neuroinflammation. Our study clearly shows that reduction in the splenic immune cell population reduced neuronal damage and CCL20 production.

Interestingly, CCL20 is a unique chemokine known to interact specifically with CC chemokine receptor 6 (CCR6) and induce chemotaxis of dendritic cells, T cells and B cells [29], all of which reside in the spleen and have the potential to promote neuroinflammation. Several lines of evidence support this hypothesis. Ohta et al. [30] have shown that CCL20 was up-regulated under normothermic conditions in a rat middle cerebral artery occlusion (MCAO) model. CCL20 is also expressed in inflamed epithelial cells [31] and in the synovial tissues of rheumatoid arthritis patients [32,33], while up-regulation of CCL20 along with other cytokines has been observed in human subjects one day after severe traumatic brain injury [34]. Furthermore, a recent study identified CCL20 as a dual-acting chemokine with the potential for inhibiting immune reactions and more importantly in attracting inflammatory effectors and activators [35]. Although a great deal of investigation has recently been done to elucidate the relationship between brain trauma and the immune system, very little is known about the function of the thymus after brain trauma. Since the thymus is the major source of mature circulating T cells, CCL20 expression in the thymus as observed in this study seems significant, although further investigation is needed to identify the specific function of thymus after TBI in adult rats.

Because CCL20-CCR6 signalling is now known to facilitate the immune response in pathological circumstances, data from the present study demonstrating up-regulated CCL20 in spleen and thymus 24 h post-LFPI likely reflects the initiation or persistence of a systemic signal that drives neural inflammation and cell death. Mouse models of autoimmune encephalomyelitis (EAE) have provided some evidence that T cells may be targeted by the splenic signal. A recent knockout study demonstrated that CCR6 modulates the infiltration of T cells into the brains of EAE-infected mice, although reduced infiltration of Treg in CCR6-/- mice was associated with increased neurological damage [36]. Despite evidence of a protective role for CCR6 activation, CCL20 signaling through CCR6 on Th1 or Th17 cells, rather than Treg cells, would be expected to promote inflammation. CCR6 is constitutively expressed in the choroid plexus of mouse and human and there are data showing that the binding of CCL20 to CCR6 on Th17 cells is critical for T cell infiltration into the CNS through the choroid plexus [37]. Indeed, T cells are well known for infiltrating the brain in neural injury models characterized by a compromised BBB. Because BBB degradation is also a critical component of TBI [19,23], peripheral CCL20 signalling may be an important initiator of T cell chemotaxis and extravasation into the brain parenchyma.

Data presented in this report also show that CCL20 was not expressed in degenerating cortical or hippocampal cell layers until 48 h after the impact. This raises the question of why cortical and hippocampal neurons expressed CCL20 at 48 h, which is 24 h after the systemic expression of the same chemokine and the neurodegeneration in the same areas of the injured brain. Although CCL20 is produced by astrocytes in response to bacterial infections [38] and EAE [39], to the best of our knowledge, ours is the first report to demonstrate neuronal expression of CCL20. One possibility is that cellular injury induces expression of CCL20 as a signal for peripheral or local immune cell recruitment to the injured site. If so, it is also likely that the neuronal cells that expressed CCL20 were in the immediate vicinity of those cells undergoing neurodegeneration. However, another possibility is that neuronal CCL20 expression is a 'tombstone' marker in cells that are beyond repair and need to be removed from the surrounding viable tissues. This latter explanation is supported by the pyknotic morphology that was observed in CCL20-expressing neurons, as well as the fact that the areas surrounding the cell bodies appeared to be devoid of tissue. The morphological analysis, anatomical localization and colocalization with FJ and NeuN protein of CCL20-positive cells strongly suggest that neurons represent the predominant cell type expressing this chemokine following TBI. Preliminary observations from this laboratory indicate downregulation of peroxysome proliferator-activated receptor γ (PPARγ) in neuronal cells (data not shown) after TBI; however, a causal role of PPARγ in regulating CCL20 signalling and/or expression in these cells remains to be established.

While results here demonstrate a link between CCL20 expression and LFPI-induced injury and indicate involvement of peripheral immune organs like the spleen in this response, further experiments are required to define the precise mechanisms by which CCL20 signalling contributes to cell death and the exact role played by spleen and thymus in inducing neuronal death. Furthermore, if CCL20 exerts direct actions on neurons, the 11 kDa protein could easily enter the CNS from the systemic circulation and promote injury even in the absence of peripheral leukocyte recruitment. If this latter scenario is the case, plasma CCL20 levels could be utilized as an important biomarker indicating the presence and severity of TBI.

## Conclusion

This study identified CCL20 as a potential novel target for anti-inflammatory therapeutic intervention. Data from this study clearly showed that LFPI-induced brain injury evoked an inflammatory reaction in the injured brain and attracted a population of activated microglia resulting in further damage of the brain. The fact that CCL20 expression is elevated in the spleen and thymus prior to its appearance in the brain, and that brain CCL20 expression is decreased in splenectomised rats provide evidence that a peripheral CCL20 signal mediates the neuropathological response to TBI. These results suggest that CCL20 plays an important role in neuroinflammation in the brain after TBI, and that peripheral CCL20 signalling promotes the secondary phase of neural injury. Future studies investigating an extended time course encompassing hours to weeks after LFPI will be critical in determining the tissue- and cell-specific origins, mechanisms, and overall effects of CCL20 signalling on TBI.

## Competing interests

The authors declare that they have no competing interests.

## Authors' contributions

SM and SSM have contributed to the conception and experimental design of the study. MD carried out most of the experimental work, analysed the data and prepared the figures of the manuscript. SR contributed to the immunostaining. SM, MD and CCL wrote the manuscript. SSM, SM and KRP reviewed and rated the manuscript. All authors have read and approved the final manuscript.
